# What is the need for adrenalectomy in patients with congenital adrenal hyperplasia in the era of CRF1/ACTH inhibitors?

**DOI:** 10.3389/fendo.2025.1693063

**Published:** 2025-11-05

**Authors:** Olga Fedorova, Henrik Falhammar

**Affiliations:** ^1^ Department of Endocrinology, Mediclinic Parkview Hospital, Mediclinic Middle East, Dubai, United Arab Emirates; ^2^ Department of Molecular Medicine and Surgery, Karolinska Institutet, Stockholm, Sweden; ^3^ Department of Endocrinology, Karolinska University Hospital, Stockholm, Sweden

**Keywords:** congenital adrenal hyperplasia, adrenalectomy, crinecerfont, atumelnant, Lu AG13909, 21-hydroxylase deficiency

## Abstract

Bilateral adrenalectomy for congenital adrenal hyperplasia (CAH) has been a historical therapeutic option for carefully selected patients who have had unsatisfactory outcomes with conventional medical management, as well as those with large adrenal tumors and hyperplasia. Rarely, adrenalectomy is performed in an undiagnosed patient with CAH due to suspicion of adrenocortical cancer, and after the surgery CAH is diagnosed. However, there are fears of increased risk of adrenal crisis and growth of adrenal rest tumors post-adrenalectomy, especially after bilateral adrenalectomy. Moreover, an adrenalectomy is a quite extensive procedure. Now with the newly approved crinecerfont (a corticotropin-releasing factor type 1 receptor (CRF1) antagonist, approved by FDA in US December 2024), and the coming phase 3 study of atumelnant (a MC2R antagonist), the commenced phase 2 study of Lu AG13909 (an anti-ACTH monoclonal antibody) as well as the preclinical studies with OMass MC2R antagonist compounds, there may be new options to decrease the size of the adrenal tumors/hyperplasia. Thus, these may be used instead of adrenalectomy. However, the cost of these new drugs may be very high so they may not become widely available, and adrenalectomy may prove to be more cost effective. This review will discuss the current use of adrenalectomy in patients with CAH and how the introduction of the new drugs may change it.

## Introduction

Congenital adrenal hyperplasia (CAH) comprises a group of autosomal recessive enzymatic disorders of adrenal steroidogenesis, most commonly 21-hydroxylase deficiency (21OHD), which accounts for about 95-99% of cases (1, 2). The second most common form of CAH is 11β-hydroxylase deficiency (11OHD) accounting for most of the non-21OHD CAH (3). In 21OHD, cortisol (and to a varying extent aldosterone) production is impaired due to *CYP21A2* gene variants, leading to cortisol deficiency, compensatory adrenocorticotropic hormone (ACTH) excess, and overproduction of steroid precursors proximal to the blocked enzymatic step, which are shunted to the production of adrenal androgens (4). The severity depends on the degree of enzyme deficiency.

Traditionally, the literature has distinguished between classic and nonclassic (NC) forms of CAH; however, current perspectives consider *CYP21A2* variants and their phenotypic presentations as part of a continuous spectrum (4). Classic CAH includes the salt-wasting (SW) form (with severe cortisol and aldosterone deficiency as well as androgen excess) and the simple-virilizing (SV) form (with cortisol deficiency and androgen excess but with milder aldosterone deficiency). NC-CAH is a milder form, typically with only slight androgen excess. NC-CAH is characterized by late-onset, partial enzyme activity and a higher prevalence (estimated prevalence ranging from 1 case per 200 people to 1 case per 1000 people) (5, 6).

Classic CAH has typically presented in the neonatal period with adrenal crisis (vomiting, weight loss, hypotension) in SW forms or signs of severe androgen excess, such as atypical genitalia in 46,XX individuals, seen in both SW and SV (7). SV has sometimes, especially in males, presented later with premature adrenarche, rapid growth velocity in childhood, adrenal or adrenal rest tumors in adulthood (8). Clinical presentations of SW and SV forms overlap, and this subtyping is often not clinically meaningful (6). However, nowadays most patients with classic CAH due to 21OHD are diagnosed in the neonatal screening programs available in most high-income and many other countries as well (4, 9).

Therapeutic strategies for patients with classic CAH aim to replace cortisol deficiency and suppress excessive androgen production with supraphysiological glucocorticoid doses (4). Adrenalectomy can occasionally be performed to improve the androgen control or avoid chronic glucocorticoid overexposure. However, there is a risk of adrenal crisis and an adrenal rest tumor growth. New pharmacological agents such as corticotropin-releasing factor type 1 receptor (CRF1) antagonists, melanocortin type 2 receptor (MC2R) (the ACTH receptor on adrenal cortical cells) antagonists and anti-ACTH monoclonal antibody aim to suppress ACTH or block its downstream effects, thereby enabling more physiological glucocorticoid dosing and minimizing the adverse effects of long-term glucocorticoid excess (6). These agents demonstrated promising reductions in 17-hydroxyprogesterone (17OHP), ACTH, androstenedione (A4) and probable decrease of adrenal hyperplasia.

The aim of this narrative review is to critically evaluate the potential of these evolving new treatment options to replace adrenalectomy in patients with classic CAH. The review is based on PubMed searches covering the period from inception to August 2025.

## Therapeutic challenges in CAH management

In contrast to other types of adrenal insufficiency, the management of CAH has two main objectives: restoring deficient hormone concentrations and controlling excess androgen production. In pediatric patients, effective suppression of adrenal androgens supports normal growth, development, and the attainment of expected adult height. In adulthood, treatment goals expand to include maintaining fertility and monitoring for long-term effects of glucocorticoid therapy (10).

Physiological replacement doses are often insufficient to adequately suppress ACTH, necessitating supraphysiologic dosing. This results in long-term complications associated with chronic glucocorticoid overexposure, including obesity, reduced final adult height, metabolic syndrome, osteoporosis, and impaired fertility (11). Even with frequent dosing of hydrocortisone, cortisol concentrations fluctuate, leading to periods of relative cortisol lack (especially overnight/early morning) that allow androgen “breakthrough,” alternating with cortisol excess that risk Cushingoid adverse effects. Thus, children and adults with CAH may cycle between androgen excess and GC excess in the course of a day (12).

Therapeutic decisions rely on both clinical findings and biochemical markers; however, the relevance and interpretation of each biomarker, such as 17OHP and A4 concentrations (and progesterone concentrations in women planning pregnancy), remain challenging due to marked circadian variability, short hydrocortisone half-life (especially in children) and interindividual variability in glucocorticoid pharmacokinetics (PK) and tissue sensitivity. Interpretation of biomarkers depends on treatment goals (e.g., growth or fertility) and it is complicated by the absence of standardized target ranges (especially in children), making it difficult to titrate glucocorticoid doses without risks of either overtreatment or hyperandrogenism (10).

In CAH, hyperandrogenism leads to menstrual irregularities, hirsutism, and infertility in females, while in males chronically elevated ACTH may result in testicular adrenal rest tumors (TARTs), which impair sperm production. TARTs are benign tumors that resemble adrenal tissue in structure and function, likely originating from adrenal cells that migrate with the gonads during fetal development. These tumors are found in up to 86% of males with CAH (13). TARTs may already be identified in childhood, with prevalence rising through puberty and adulthood, and have even been documented at autopsy in patients with CAH younger than eight weeks. Elevated ACTH is considered the major pathogenic factor of TARTs, and they are most often described in patients with poor hormonal control (14). Nonetheless, TARTs do not develop in all patients with poor hormonal control and can also appear in well-controlled individuals, suggesting that the timing of ACTH elevation might play an important role. Interestingly, TARTs have also been reported in acquired conditions with marked ACTH excess, including Cushing and Addison disease as well as Nelson syndrome, highlighting the role of prolonged ACTH exposure in their development (15).

TARTs can be identified by ultrasound or magnetic resonance imaging (MRI) and often shrink with optimized glucocorticoid therapy if treated early before fibrosis has developed. Surgery does not restore fertility but may occasionally be performed due to local symptoms (16). In women, ovarian adrenal rest tumors (OARTs) have historically been considered rare, with fewer than 20 cases reported (17). However, recent findings using ^11^C-metomidate positron emission tomography/computed tomography (PET/CT) imaging (18) suggest that OARTs may be more prevalent than previously believed, particularly in women who require unusually high doses of glucocorticoids for androgen suppression.

Adrenal crisis remains a frequent and potentially fatal complication in patients with CAH. A nationwide population-based study by Falhammar et al. (19) showed that adrenal crisis is the leading cause of death in patients with CAH. Adrenal crisis is particularly common in children with CAH aged 1 to 5 years (20).

## Adrenal tumors in CAH

Adrenal tumors are typically defined as lesions measuring 10 mm or more (21). However, some have used thresholds of 5 mm; e.g., Jaresch et al. (22) reported an 80% prevalence of adrenal tumors in 22 patients with CAH based on computer tomography (CT) scans, using a cutoff of >5 mm to define a tumor. Notably, half of the identified lesions measured only 5 to 9 mm, suggesting that some cases of macronodular hyperplasia may have been misclassified as tumors (22).

It is well known that increased ACTH concentrations act as a growth factor on adrenal cells resulting in large adrenal glands. It remains unclear whether ACTH hypersecretion directly contributes to tumor growth, but it may help explain the high prevalence of myelolipomas and adrenal adenomas in patients with CAH (23).

Myelolipoma, a benign tumor composed of adipose and hematopoietic tissue, is the most frequent histological type of adrenal tumors in patients with CAH (24). A meta-analysis of Nermoen at el (23). showed that 28.1% of patients with CAH and genetically confirmed 21OHD had at least one adrenal tumor. Thus, patients with CAH have more than 30 times increased risk of having adrenal tumors compared to the general population. Tumors occurred more frequently in males and in individuals with poorly controlled disease (23). Hagiwara et al. (25) found no overexpression of ACTH or androgen receptors in a giant myelolipoma in a woman with CAH. In contrast, Almeida et al. (26) reported elevated expression of ACTH (melanocortin 2 receptors) and androgen receptors in giant bilateral myelolipomas from patients with CAH. ACTH is nowadays considered to be one of the main drivers for myelolipomas but not the only one (24). Given the high prevalence of adrenal tumors among patients with CAH, it has been proposed that adrenal imaging may be performed once in adulthood and potentially repeated every 5 to 10 years based on initial findings (23). Imaging is especially warranted in the presence of abdominal pain, as myelolipomas may present with this symptom (23). Currently, no reliable biomarkers exist to guide the frequency of imaging. Tumor size in CAH has been associated with elevated 17OHP concentrations (27), but 11-oxo-androgens (and 21-deoxycortisol) correlate better with adrenal size (28). It should be noted that 17OHP concentrations have been correlated with adrenal tumor size also in patients not affected by CAH, thus patients with large or bilateral adrenal tumors where 17OHP concentrations have been measured can be misdiagnosed as CAH if no genetic confirmation has been done (29). However, prospective longitudinal studies using comprehensive steroid profiling are still needed.

In contrast, rarely undiagnosed CAH can underlie adrenal tumors even in elderly patients, with some cases initially suspected to be adrenocortical carcinoma (ACC) due to their size and imaging features (27–30). Adrenalectomy performed without recognizing underlying CAH may precipitate an adrenal crisis due to impaired cortisol response. International guidelines currently do not support routine screening for CAH in patients with adrenal incidentalomas (21). A meta-analysis demonstrated that screening with 17OHP concentrations appears to lack specificity in the setting of adrenal incidentalomas in adults, especially if <60 nmol/L (8). Nonetheless, the same meta-analysis showed that 0.8–5.9% of all patients with adrenal incidentalomas had CAH (8). Thus, in all patients with an adrenal tumor, CAH could be considered (31).

## Adrenalectomy in CAH

Unilateral or bilateral adrenalectomy has been considered as an approach to long-term management of classic CAH to limit adrenal androgens, particularly in patients with severe hyperandrogenism unresponsive to optimized medical therapy or in those with large adrenal tumors causing mass effect or diagnostic uncertainty (32). Surgical removal of adrenal glands allows glucocorticoid replacement at physiologic doses, eliminating the need for ACTH suppression and potentially improving androgen control without the adverse effects of high-dose glucocorticoids. In a meta-analysis on bilateral adrenalectomy in patients with CAH by MacKay et al. (33) 48 patients with CAH and bilateral adrenalectomy were described. Of these 34 (71%) described symptomatic improvement after bilateral adrenalectomy. Reported benefits included reduction in signs of hyperandrogenism, resumption of regular menses or onset of menarche, acceleration of growth rate, and amelioration of Cushingoid features, particularly decreased body weight. Ten case reports documented patient or parental satisfaction, often related to enhanced body image. Of the five patients undergoing bilateral adrenalectomy for treatment of hypertension (all with CAH due to 11OHD), three achieved remission and discontinued antihypertensive therapy (34, 35). Three women underwent bilateral adrenalectomy for treatment of primary infertility; all subsequently conceived (33).

According to this meta-analysis, the most common indication for bilateral adrenalectomy was the presence of hyperandrogenic or virilizing symptoms (52%), followed by iatrogenic Cushing syndrome (21%) (33). In 29% of cases, both hyperandrogenism and Cushing syndrome were indications for the surgery. Other indications included infertility (6%), large adrenal tumors such as myelolipomas or adenomas causing symptoms or raising suspicion of malignancy (17%), and the management of refractory hypertension.

However, bilateral adrenalectomy can also result in adverse events. In the meta-analysis 5 cases (10%) reported short-term complications (including episodes of transient ischemic attack without residual neurologic deficit in 1 patient, a postoperative hypertensive crisis, persistent sinus tachycardia, superficial wound infection and postoperative ileus with hypotension) and 13 cases (27%) – long-term adverse outcomes (see below) (33).

### Adrenal crisis

Adrenal crisis is a life-threatening event (36) and represents the leading cause of mortality in individuals with CAH, accounting for 42–58% of deaths (19). Among patients receiving treatment for CAH, approximately one-third experience an adrenal crisis by adolescence, with the incidence rising to 57% in adulthood (37, 38).

The incidence of adrenal crisis in patients with CAH has been estimated to 5.8 per 100 patient-years (37), while MacKay et al. (33) reported a higher rate of 14.8 adrenal crises per 100 patient-years following bilateral adrenalectomy. In a previous systematic review on 18 patients with CAH and bilateral adrenalectomy, postoperative adrenal crises occurred at similar rates in both adults and children; however, the pediatric cases tended to be more severe, including one child who required long-term antiepileptic therapy following a hypoglycemic seizure (39). These findings may support postponing bilateral adrenalectomy until late adolescence or adulthood to reduce the risk of severe adrenal crises and their complications.

Elective bilateral adrenalectomy carries a recognized risk of postoperative morbidity and mortality, and patients with a history of poor adherence to medical therapy are generally unsuitable candidates. In such individuals, the likelihood of complications may outweigh the expected benefits. Even so, the possibility of adrenal crisis after bilateral adrenalectomy should not, on its own, rule out this intervention for every patient (33).

### Adrenal rest tumors

Bilateral adrenalectomy may not totally remove hyperandrogenemia owing to the potential development of adrenal rest tumors in the testes (39), ovaries (40), or retroperitoneum (41) due to elevated ACTH concentrations. ACTH, as previously noted, is thought to play a central role in the growth of TART. A case of bilateral adrenalectomy in a patient with CAH has been reported, where ^11^C-metomidate PET/CT was performed to investigate recurrent hyperandrogenism. The patient was found to have OART and retroperitoneal adrenal rest tumors (18).

### Other complications

Two children with CAH and bilateral adrenalectomy developed severe hyperpigmentation and pituitary microadenomas after bilateral adrenalectomy (one of them demonstrated history of poor adherence to therapy). To date, no studies have described pituitary macroadenomas causing optic nerve or chiasmal compression in CAH (33, 42), a feature sometimes seen in Nelson syndrome after bilateral adrenalectomy for Cushing disease (43).

For these reasons, despite initial enthusiasm driven by short-term success, adrenalectomy has largely fallen out of favor due to long-term complications. The Endocrine Society Clinical Practice Guidelines suggest that in patients with CAH bilateral adrenalectomy should not be performed (2). This is too conservative we believe. Adrenal crises are mainly seen in younger patients, supporting the idea of delaying bilateral adrenalectomy until adulthood when possible (33). Prior poor adherence to glucocorticoid replacement was common among those who developed adrenal crises and ectopic tissue activation, however, if the patient demonstrates good adherence the last year or so, in our opinion, we could still do bilateral adrenalectomy in selected cases.

## Emerging pharmacologic alternatives to adrenalectomy

**Figure 1:**
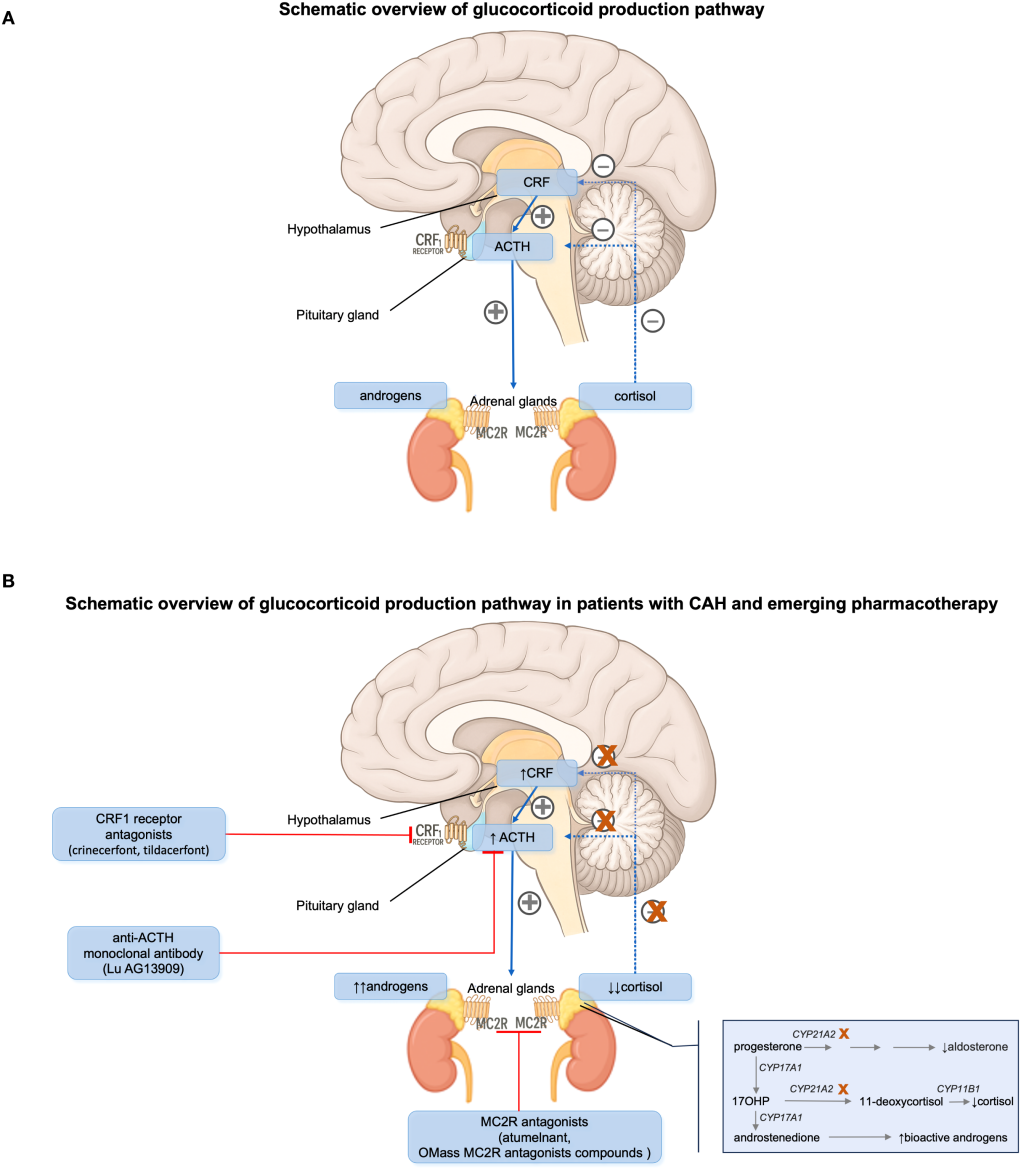
**(A)** Hypothalamic-pituitary-adrenal (HPA) axis and its hormonal regulation in normal humans. **(B)** HPA axis and its hormonal regulation in patients with congenital adrenal hyperplasia (CAH) due to 21-hydroxylase deficiency. ACTH, adrenocorticotropic hormone. CRF, corticotropin-releasing factor. MC2R, melanocortin type 2 receptor.

Recent developments in targeted therapies for CAH focus on reducing ACTH stimulation and thereby limiting androgen excess and adrenal hyperplasia. These agents aim to replace or augment traditional glucocorticoid therapy while minimizing adverse effects (**Table 1**, **Figure 1**).

**Table 1:** Comparison of emerging pharmacological treatment for patients with congenital adrenal hyperplasia (CAH).

Drug	Mechanism of action	Development stage	Hormonal effect	Adrenal/TART size effect
Crinecerfont (44–49)	CRF1 antagonist (reduces ACTH secretion from pituitary gland)	Approved (FDA, Dec 2024) – Phase 3 trials completed	↓ ACTH, 17OHP, A4; ↓ Testosterone in females; ↓ A4:T ratio in males significant ↓ glucocorticoid dose	Theoretical potential for TART/adrenal size reduction
Tildacerfont (50, 51)	CRF1 antagonist (reduces ACTH secretion from pituitary gland)	Phase 2 completed; Phase 2b trials terminated early (did not meet their primary endpoints) and drug development ceased	↓ ACTH, 17OHP and, A4	TART volume reduction observed in individual cases
Atumelnant (52–60)	MC2R antagonist (blocks receptor for ACTH on adrenal cortical cells)	Phase 2 ongoing; Phase 3 study is being initiated	↓ A4, 17OHP and 11-oxo-androgens	↓ Adrenal gland volume (median −13.7%, range −36% to 49%) after 12 weeks; 10/12 patients had volume reduction in one or both glands
Lu AG13909 (61–63)	Selective monoclonal antibody that neutralizes ACTH	Phase 1 completed; Phase 2 recruiting	↓ A4 and 17OHP	May reduce adrenal hyperplasia/tumors (needs confirmation)
OMass MC2R antagonist compounds (64)	High-affinity MC2R antagonists with prolonged receptor residence time – insurmountable antagonism	Preclinical + first-in-human enabling studies	↑residence time and insurmountability *in vitro* ↓ plasma corticosterone, prevented ACTH-induced adrenal hyperplasia, and mitigated body-weight loss in a rat model	Possible, but no human data yet

11-oxo-androgens, 11-oxygenated androgens.

17OHP, 17-hydroxyprogesterone.

A4, androstenedione.

A4T ratio, androstenedione-to-testosterone ratio.

ACTH, adrenocorticotropic hormone.

CAH, congenital adrenal hyperplasia.

CRF1, corticotropin-releasing factor receptor type 1.

FDA, U.S. Food and Drug Administration.

MC2R, melanocortin type 2 receptor.

TART, testicular adrenal rest tumor. Arrow down (↓), decreased. Arrow up (↑), increased.

## Crinecerfont

One promising new strategy for reducing adrenal androgen overproduction through a glucocorticoid-independent mechanism is CRF1 antagonism to reduce ACTH secretion, thus potentially allowing for physiological glucocorticoid dosing (44).

Crinecerfont is a novel orally administered CRF1 antagonist, approved by the U.S. Food and Drug Administration (FDA) in December 2024 for use alongside glucocorticoid therapy to help manage androgen concentrations in both pediatric (aged 4 and above) and adult patients with classic CAH (44, 45). Phase 2 trials in adults (46) and adolescents (47) demonstrated that crinecerfont significantly lowered ACTH, 17OHP and A4 concentrations after 14 days of open-label treatment, justifying that CRF1 antagonism has therapeutic value in CAH. Additionally, reductions in testosterone concentrations in females and in the androstenedione-to-testosterone ratio in males were also observed.

In two phase 3 trials, crinecerfont led to substantial reductions in androstenedione and 17OHP concentrations by week 4 compared to placebo. In adults, A4 decreased by -299 ng/dL (-10 nmol/L) *vs*. +45.5 ng/dL (+1.6 nmol/L) with placebo, and 17OHP by -5594 ng/dL (-170 nmol/L) *vs*. -156 ng/dL (-4.7 nmol/L) (44). In children, A4 declined by -197 ng/dL (-6.9 nmol/L) *vs*. +71 ng/dL (+2.5 nmol/L), and 17OHP by -5865 ng/dL (-178 nmol/L) *vs*. +556 ng/dL (+16.8 nmol/L) (48). Crinecerfont allowed for significantly greater glucocorticoid dose reductions at week 24 in adults (-27.3% *vs*. -10.3%) and at week 28 in children (-18.0% *vs*. +5.6%), while A4 concentrations were maintained within 120% of baseline. A higher proportion of participants achieved physiologic glucocorticoid dosing as compared to placebo (≤11 mg/m²/day): 63% *vs*. 18% at week 24 in adults, and 30% *vs*. 0% at week 28 in children (44, 45). Fatigue and headache were the most common adverse events.

The primary goal of the adult crinecerfont study was to support the use of physiological doses of glucocorticoids. If this outcome is sustained and the reduction in glucocorticoid use proves to be clinically meaningful, crinecerfont could help reduce long-term risks such as cardiometabolic complications and osteoporosis with fractures. Clinically, crinecerfont may be attractive also for short-term use to suppress excessive androgen production when hormonal control has deteriorated for some reason, without overtreatment with glucocorticoids. The drug might also benefit selected patients who consistently show elevated morning androgen concentrations, although for these individuals, modified-release hydrocortisone formulations may offer a more physiological approach (49). It remains unclear whether crinecerfont can effectively treat testicular adrenal rest tumors or benign adrenal tumors in patients with CAH but theoretically it may work.

## Tildacerfont

Tildacerfont is a selective, nonsteroidal, second-generation CRF1 antagonist that binds pituitary CRF1 to reduce ACTH secretion. In a Phase 2 study (study 201), tildacerfont reduced ACTH, 17OHP, and A4 concentrations in adults with poorly controlled classic CAH, though no clear dose-response relationship was observed (50). Among participants with poor disease control despite supraphysiologic glucocorticoid doses, mean reductions were 38.3% for 17OHP, 24.2% for A4, and 59.4% for ACTH. In participants with good disease control at baseline, tildacerfont did not further reduce ACTH or A4 concentrations (already within the normal range), but 17OHP concentrations declined in those with elevated baseline values (50).

In a second Phase 2 trial (study 202), tildacerfont 400 mg/day for 10–12 weeks resulted in maximum mean reductions of 84% in ACTH, 82% in 17OHP, and 79% in A4 concentrations. However, the phase 2b studies, which were randomized controlled trials, did not meet their primary endpoints and were terminated early in 2024 (51). Yet, they provided valuable data for future CAH research. A trend toward greater A4 reduction was noted with higher twice-daily doses (45).

Tildacerfont was generally well tolerated. Adverse events occurred in 53.6% of participants, most commonly headache (7.1%) and upper respiratory tract infection (7.1%), and were predominantly mild (50).

In study 201 a patient treated with tildacerfont experienced a 23% reduction in TART volume after 6 weeks. This decrease in TART size occurred together with an 87% reduction in ACTH concentration, suggesting a direct link between lowering ACTH and TART regression. In study 202 one patient with CAH showed no change in either TART volume or ACTH concentrations. By contrast, another patient had four measurable TARTs at baseline, all of which completely regressed and were no longer detectable after treatment (50). No data on adrenal hyperplasia and tumor volume exist.

## Atumelnant

Another potential strategy to reduce excess adrenal androgens is via blocking the MC2R, the receptor for ACTH on adrenal cortical cells. Preclinical studies in rodents demonstrated that antagonizing this receptor led to a dose-dependent suppression of plasma corticosterone and reversal of adrenal gland hypertrophy. These findings provided the rationale for initiating phase 1 dose-escalation trials of atumelnant, an oral MC2R antagonist, in healthy volunteers (45, 52). In these studies, atumelnant (10–80 mg daily) was well tolerated and showed a dose-dependent suppression of morning serum cortisol, A4 and aldosterone concentrations, along with a blunted response to ACTH stimulation (53).

Following these encouraging results, atumelnant advanced to phase 2 trials for both classic CAH (54) and ACTH-dependent Cushing syndrome.

The CAH trial enrolled adult patients with CAH who had been on a stable glucocorticoid regimen for at least six months. Participants received once-daily oral atumelnant for a 12-week treatment period. Preliminary data from the first four patients treated with the 80 mg dose showed a rapid and substantial decrease in A4 concentrations within two weeks, with reductions ranging from 74% to 99%, which were sustained throughout the treatment period. A similar pattern was observed with 17OHP concentrations, where concentrations dropped by 68% to more than 99% (55). In the full study cohort (40 mg, n=11; 80 mg, n=11; 120 mg, n=6), once-daily atumelnant produced rapid (by week 2) and sustained reductions in 11-oxo-androgens (11β-hydroxyandrostenedione and 11-ketotestosterone), A4, and 17OHP concentrations, with the magnitude of reduction increasing with dose (56). In addition to biochemical improvements, interim results demonstrated adrenal volume reductions on standardized MRI in 12 patients with classic CAH. Baseline median (range) total adrenal volume was 19.7 mL (10.2 – 943.6 mL). After treatment, the median (range) change was -4.3 mL (-77.5 to 9.1 mL), corresponding to -13.7% (-36% to 49%), with reductions in at least one adrenal gland in 10 of 12 patients (57). Importantly, no serious or treatment-related adverse events were reported, and the drug was generally well tolerated (55).

Further data are required to establish its long-term efficacy and safety profile. According to Crinetics’ 2025 report, a Phase 3 study named CALM–CAH is being initiated, aiming to demonstrate normalization of A4 concentrations in adults with CAH under physiological glucocorticoid replacement. Additionally, industry insights suggest a pediatric Phase 2/3 program is expected later in 2025 (58, 59). In August 2025 atumelnant received FDA Orphan Drug Designation for the treatment of patients with CAH, underscoring recognition of its potential clinical value (60).

## Lu AG13909

Lu AG13909 is a selective, high-affinity monoclonal antibody that neutralizes ACTH. In preclinical studies involving rodents and cynomolgus monkeys, administration of Lu AG13909 led to dose-dependent reductions in plasma corticosterone and cortisol, respectively. Importantly, no adverse effects were reported following intravenous administration every two weeks for six months in cynomolgus monkeys (61).

An open-label, phase 1 dose-escalation trial has recently finished and assessed the safety, tolerability, pharmacokinetics and pharmacodynamics of Lu AG13909 in adults with CAH (45, 62). The study included male and female participants aged 18 to 70 years who were on stable glucocorticoid and mineralocorticoid therapy and had morning 17OHP concentrations exceeding four times the upper limit of normal. Each participant was scheduled to receive up to six intravenous doses of Lu AG13909 at intervals of 28 to 35 days, with progressive dose increases. In total 11 adult patients were recruited. The drug was well tolerated. Morning 17OHP and A4 concentrations were reduced at 24h after infusion by 90.5-98.7% and 66.3-89.3%, respectively, across all dose levels (63).

A phase 2 study has just started recruiting patients during summer 2025. If effective, Lu AG13909 could provide sustained androgen control and may also reduce adrenal hyperplasia and tumors.

Compensatory elevations of plasma ACTH may occur with atumelnant and Lu AG13909 treatment, which can theoretically contribute to pituitary hyperplasia, although no such findings have been reported to date.

## OMass MC2R antagonist compounds

A new series of selective, high-affinity MC2R antagonists was designed by OMass with prolonged receptor residence time, optimized to ensure insurmountable antagonism, the ability to maintain MCR2 blockade even in the presence of very high ACTH concentrations. The compounds were tested *in vitro* and *in vivo* using acute ACTH 1–24 challenges, mimicking the surges seen in patients with CAH (64).

The lead candidate, Compound 5, demonstrated a residence time of 165 minutes at human MC2R (*vs*. 1.7 minutes for atumelnant) and achieved 87% insurmountability *in vitro*. *In vivo*, oral administration of Compound 5 inhibited ACTH-stimulated corticosterone production more effectively than atumelnant, with a half-maximal effective concentration of 5.0 ng/mL and maximal suppression of 94% (*vs*. 256.1 ng/mL and 70.2% for atumelnant) (64).

In a rat model of chronic ACTH infusion, Compound 5 dose-dependently reduced plasma corticosterone, prevented ACTH-induced adrenal hyperplasia, and mitigated body-weight loss. By exploiting prolonged receptor residence time, these antagonists can maintain full MC2R inhibition despite extreme ACTH concentrations, potentially improving hormonal control in patients with 21OHD without requiring supraphysiologic glucocorticoid dosing. Compound 5 has progressed to first-in-human studies (64). In theory this drug could have the potential to reduce adrenal hyperplasia and tumor volume.

## Discussion

The role of adrenalectomy in CAH remains incompletely defined, with most evidence derived from small series and case reports. Long-term data on fertility, metabolic outcomes, and quality of life are limited, and publication bias may overrepresent successful cases. The lack of standardized endpoints further complicates comparisons across studies.

Our review is limited by the quality and heterogeneity of the available literature on emerging therapeutic options, as well as the preliminary nature of data on these pharmacological agents. Most evidence is limited to early-phase trials of relatively short duration, and while results are promising, long-term safety, efficacy and real-world effectiveness remain uncertain.

Practical limitations should also be acknowledged, e.g., drug-drug interaction between tildacerfont and dexamethasone where studies have shown that co-administration leads to approximately double the dexamethasone exposure, likely via CYP3A4, necessitating the use of alternative glucocorticoids.

Global accessibility of new medications is limited by cost. Crinecerfont’s U.S. list price is approximately $460,000 annually for adults and $230,000 for pediatric patients under 20 kg. In resource-limited settings, adrenalectomy may still be considered a more cost-effective option, especially where modern therapies are unavailable or unaffordable. Cost-effectiveness analyses will therefore be essential to define the place of these agents in long-term management.

## Conclusion

The management of CAH is entering a new therapeutic era. While bilateral adrenalectomy has historically served as a last-resort intervention for patients with refractory hyperandrogenism or large adrenal tumors, it is an extensive and irreversible procedure associated with increased risk of adrenal crisis, and possible activation of ectopic adrenal rest tissue. For these reasons, current clinical guidelines do not support its routine use.

The development of therapies targeting the hypothalamic-pituitary-adrenal (HPA) axis presents promising alternatives. CRF1 antagonists (crinecerfont, approved by FDA in December 2024), MC2R antagonists (atumelnant, soon phase 3; OMass MC2R antagonist compounds, waiting phase 1), and ACTH-neutralizing monoclonal antibodies (Lu AG13909, ongoing phase 2) have demonstrated the potential to suppress/block ACTH, reduce adrenal androgen excess, and improve disease control while preserving adrenal integrity and enabling more physiologic glucocorticoid dosing. These agents might also help reduce adrenal hyperplasia and tumor size. Combination regimens – such as the use of a CRF1 antagonist together with an ACTH-targeting agent, or maybe modified released hydrocortisone – may represent a potential future strategy for improving disease control in severe cases, though this approach remains theoretical at present.

Despite current limitations, the therapeutic trajectory is clear. As these pharmacologic agents become more widely available and integrated into clinical practice, the need for adrenalectomy is expected to decline. In most cases, individualized, targeted medical therapy could offer a safer, more flexible, and effective strategy for managing CAH – reserving surgery only for rare, exceptional scenarios such as large tumors causing mass effect or treatment-refractory disease in low-resource settings.
